# Risk stratification by long non‐coding RNAs profiling in COVID‐19 patients

**DOI:** 10.1111/jcmm.16444

**Published:** 2021-03-23

**Authors:** Jie Cheng, Xiang Zhou, Weijun Feng, Min Jia, Xinlu Zhang, Taixue An, Minyuan Luan, Yi Pan, Shu Zhang, Zhaoming Zhou, Lei Wen, Yun Sun, Cheng Zhou

**Affiliations:** ^1^ Center for Reproductive Medicine Ren Ji Hospital School of Medicine Shanghai Jiao Tong University Shanghai China; ^2^ Shanghai Key Laboratory for Assisted Reproduction and Reproductive Genetics Shanghai China; ^3^ Department of Anesthesiology General Hospital of Central Theater Command of PLA Wuhan China; ^4^ Institute of Pediatrics Children’s Hospital of Fudan University the Shanghai Key Laboratory of Medical Epigenetics International Co‐laboratory of Medical Epigenetics and Metabolism Ministry of Science and Technology Institutes of Biomedical Sciences Fudan University Shanghai China; ^5^ Department of Radiation Oncology Fudan University Shanghai Cancer Center Fudan University Shanghai China; ^6^ Department of Cardiology Nanfang Hospital Southern Medical University Guangzhou China; ^7^ Department of Laboratory Medicine Nanfang Hospital Southern Medical University Guangzhou China; ^8^ Organ Transplantation Center the Affiliated Hospital of Qingdao University Qingdao China; ^9^ Division of Functional Genome Analysis German Cancer Research Center (DKFZ) Heidelberg Germany; ^10^ Faculty of Medicine Heidelberg Heidelberg University Heidelberg Germany; ^11^ Department of Gynecological Oncology Fudan University Shanghai Cancer Center Shanghai China; ^12^ Department of Radiation Medicine School of Public Health Southern Medical University Guangzhou China; ^13^ Department of Oncology Guangdong Sanjiu Brain Hospital Guangzhou China; ^14^ Department of Radiation Oncology Nanfang Hospital Southern Medical University Guangzhou China

**Keywords:** COVID‐19, lncRNA, pulmonary injury, RNA‐seq, transcriptome

## Abstract

Coronavirus disease 2019 (COVID‐19), caused by severe acute respiratory syndrome coronavirus 2 (SARS‐CoV‐2), has become a global pandemic worldwide. Long non‐coding RNAs (lncRNAs) are a subclass of endogenous, non‐protein‐coding RNA, which lacks an open reading frame and is more than 200 nucleotides in length. However, the functions for lncRNAs in COVID‐19 have not been unravelled. The present study aimed at identifying the related lncRNAs based on RNA sequencing of peripheral blood mononuclear cells from patients with SARS‐CoV‐2 infection as well as health individuals. Overall, 17 severe, 12 non‐severe patients and 10 healthy controls were enrolled in this study. Firstly, we reported some altered lncRNAs between severe, non‐severe COVID‐19 patients and healthy controls. Next, we developed a 7‐lncRNA panel with a good differential ability between severe and non‐severe COVID‐19 patients using least absolute shrinkage and selection operator regression. Finally, we observed that COVID‐19 is a heterogeneous disease among which severe COVID‐19 patients have two subtypes with similar risk score and immune score based on lncRNA panel using iCluster algorithm. As the roles of lncRNAs in COVID‐19 have not yet been fully identified and understood, our analysis should provide valuable resource and information for the future studies.

## 1 INTRODUCTION

Coronavirus disease 2019 (COVID‐19), caused by severe acute respiratory syndrome coronavirus 2 (SARS‐CoV‐2), has become a global pandemic, with over 13 million cases and considerably growing death worldwide. According to the 7th edition of Clinical Guidance for COVID‐19 Pneumonia Diagnosis and Treatment of China, COVID‐19 patients are categorized into four groups: mild, moderate, severe and critically severe on the basis of clinical symptoms and related medical examinations.^1^ Approximately 20% of patients with COVID‐19 develop severe disease warranting hospitalization, and approximately 5% require intensive care.^2^ Besides dyspnoea, hypoxaemia, acute respiratory distress and lymphopenia, cytokine release syndrome (CRS) is an important clinical features in patients at severe stage.^3^

Previous transcriptomic studies about COVID‐19 have focussed on the analysis of protein‐coding transcripts, using mRNA expression profiling to characterize the patterns and potential functional roles of their translated proteins.^4^ However, studies revealed that only less than 3% of the human genome is believed to be coding regions.^5^, ^6^ The rest is called junk DNA among which lncRNAs are a group of non‐coding RNAs with more than 200 nucleotides in length. The development of next generation sequencing technology has greatly shed light on the function of lncRNAs in the biological process of different disease. Besides cancer, studies have highlighted the role of lncRNAs in other disease, like immune disease.^7^, ^8^ Although some studies investigated the altered lncRNAs between COVID‐19 patients with healthy control, no related studies have investigated the lncRNA expression pattern based on the severity of COVID‐19 patients so far.^9^, ^10^ The functions for lncRNAs in COVID‐19 have not been unravelled.

The present study aimed at identifying the target lncRNAs based on peripheral blood mononuclear cells (PBMCs) from patients with SARS‐CoV‐2 infection as well as health individuals. Particular attention was paid to differential regulations of lncRNAS between severe and non‐severe symptom groups in terms of lncRNA expression pattern. Our findings from the screening of lncRNA biomarkers are expected to improve the understanding of COVID‐19 subtypes and may facilitate the further exploration of diagnosis, prognosis and even therapeutic strategies for this devastating disease.

## 2 MATERIALS AND METHODS

### 2.1 Patients and specimen collection

We collected blood from 29 patients enrolled in local hospital from March to April 2020 after written informed consent from patients. Eligibility criteria for moderate or severe patients were based on the 7th guideline. Briefly, moderate patients were with fever and respiratory symptoms. Radiologic assessments found signs of pneumonia. Severe type were patients meet any of the following criteria: (1) Shortness of breath, RR>=30 times/min; (2) Oxygen saturation <=93% at rest; (3) Alveolar oxygen partial pressure/fraction of inspiration O2 (PaO2/FiO2) <=300 mm Hg. (4) CT chest imaging shows that lung damage develops significantly within 24‐48 h. Critically severe type was patients meet any of the following criteria: (1) Respiratory failure requiring mechanical ventilation; (2) Signs of septic shock; (3) Multiple organ failure requiring ICU admission. For controls, blood was collected from 10 healthy adult donors after written informed consent. All donors were consented for genetic research. The use of human samples in this study has been ethically approved by the hospital ethics committee. Written informed consent was obtained from patients and healthy individuals before sample and data collection. The study was conducted to the principles of the Declaration of Helsinki. Lymphocyte subtyping and serum inflammatory indictor determined assay was performed at medical laboratory of hospital.

### 2.2 Temporal RNA transcript isolation, sequencing and processing

Human PBMCs were isolated by centrifugation. Peripheral blood was layered and centrifuged at 950 g for 30 min. After isolation on a Ficoll‐Histopaque layer (Sigma Aldrich, Italy), the plasma was carefully removed and the PBMC cells were transferred to falcon tubes for RNA extraction. Samples were homogenized and total RNA was isolated by TRIzol® reagent (Invitrogen by Life Technologies, NY, USA) following the manufacturer's protocol. RNAs were quantified using a Nanodrop ND‐100 Spectrophotometer (Nanodrop Technologies, Wilmington, USA) with RNAs of a 260:280 ratio ≥1.5 and an RNA integrity number ≥8 was deep sequenced. Sequencing libraries were prepared with the Illumina TruSeq Stranded RNA Library Prep, version 2, Protocol D, using 500ng total RNA (Illumina, USA). The qualities of the libraries were assessed by 2100 Bioanalyzer with a DNA1000 assay. Libraries were quantified by qPCR using the KAPA Library Quantification kit for Illumina sequencing platforms. RNA processing has conducted by Illumina NextSeq 500 Sequencing. FastQ files were generated from.bcl files produced by Illumina NextSeq sequencer by llumina bcl2fastq2.

### 2.3 Data pre‐processing and screening of differentially expressed lncRNAs

In order to identify the biological significance of each probe, the comprehensive gene annotation files were obtained from GENCODE in GTF format. The GENCODE annotation was the default gene annotation displayed in the Ensembl Genome Browser. The transcripts with a length of more than 200 nucleotides and those labelled as ‘non_coding’, ‘processed_transcript’, ‘lincRNA’, ‘retained_intron’, ‘antisense’, ‘sense overlapping’, ‘sense_intronic’ and ‘bidirectional_promoter_lncrna’ were considered as ‘lncRNAs’. Finally, 2511 expressed lncRNAs were annotated. The Affymetrix probe level data were obtained by reading the CEL files using the ReadAffy function of the Affy R package, and then, the raw data were pre‐processed (background correction, normalization and summary expression computation). Empirical Analysis of Digital Gene Expression Data in R (edger) package (http://bioconductor.org/packages/release/bioc/html/edgeR.html) was applied to explore the differentially expressed lncRNAs (DELncRNAs) between three groups using the criteria of *P*‐value <0.05 and |log2 (fold change) | >= 1.

### 2.4 Hierarchical cluster analysis and Principal Component Analysis

To investigate the relationships among the severity of patients, hierarchical cluster analysis and PCA were performed for all patients. Total 39 patients, including 12 non‐severe patients, 17 severe patients and 10 normal patients, were embedded in this step. Patients with similarly expressed lncRNAs tended to close up in hierarchical cluster analysis and PCA. We carried out the hierarchical cluster analysis using flashclust function in WGCNA R package and PCA using factoextra R packages.^11^

### 2.5 Construction of the COVID‐19 risk score

To further investigate the prognosis of COVID‐19 patients, we developed a prognosis risk score model using LASSO (Least Absolute Shrinkage and Selection Operator) regression. We selected non‐severe patients and severe patients. All lncRNAs are included. We divided patients into training set (50% patients) and test set 1 (50% patients) randomly. LASSO regression was carried out on training set and test sets ten thousand times. To assess the performance of each model, AUC (Area under curve) of the ROC (receiver operating characteristic curve) was calculated for each model. We sum square of AUC (training set) and square of AUC (test set) as the performance index of each model. We selected the model with max performance index as the best model. Seven lncRNAs were finally confirmed. The risk score for predicting severity of COVID‐19 patients was calculated based on the expression levels and the corresponding regression coefficients of the 7 lncRNAs. The equation for the risk score of the 7‐lncRNA signature was shown asfollows: risk score = 5.78‐34.59*AC010904.2‐1.05*AC012065.4‐0.00063*AL365203.2‐0.867*AC010175.1‐0.021*LINC00562‐0.0093*AC010536.1‐1.05* AP005671.1.

### 2.6 Integrative clustering using iCluster algorithm

COVID‐19 is a complex and serious disease. Different patients have quite different treatment responses and prognoses. To give patients individual treatment, we identify the subtypes of COVID‐19 patients. First, we carried out WGCNA (weighted gene correlation network analysis) algorithm for all lncRNAs of non‐severe and severe patients using default parameters. We used dynamic tree cutting method to identify the lncRNAs co‐expression modules. The co‐expression modules were assigned to different colours for visualization. We calculated the correlation of co‐expression modules and seriousness. We selected lncRNAs co‐expression modules which were significantly correlated with seriousness. Next, we implemented iCluster algorithm for the lncRNAs modules we selected. We chose the k where the curve of percent explained variation levels off. The number of the clusters is k + 1.

### 2.7 Assessment of patients’ immune status

To figure out whether immune statuses of different iCluster patients are different, we assessed the immune status of patients using GSVA R package.^12^ First, we screened the immune indices that were significant different between non‐severe and severe COVID‐19 patients using Wilcoxon's test. Immune indices which p‐value less than 0.01 were selected. Next, we screened gene signatures of these immune indices using Boruta R package.^13^ Finally, we used GSVA algorithm to assess the immune score based on the gene signatures.

### 2.8 Model selection

The optimal combination of clusters was determined minimizing a Bayesian Information Criterion (BIC). An ‘elbow’ point was noted at K = 3, beyond which the BIC kept increasing, and thus, the 3‐class solution was chosen. Figure S3C shows that the results were highly comparable for individual unsupervised clustering versus integrative clustering, indicating that the iCluster groupings represented the combined information of all platforms and lacked bias to a particular data type. To compare the resultant iCluster groupings to the molecular subclasses developed by Hoshida,^14^ we assigned each of our patients to one of the three Hoshida subclasses using their transcriptional predictors. We found strong concordance between the iClusters and the Hoshida subclasses.

### 2.9 Statistical analysis

R language software was used to test for differences in means between specific severe and non‐severe groups. The null hypothesis of no differences in means was tested using a two‐tailed t test with a *P*‐value < 0.05 deemed as significant.

## 3 RESULTS

### 3.1 Patients information

All COVID‐19 patients were randomly recruited, and among those, 12 patients had mild or moderate symptoms, accounting for 45.2%, whereas 17 patients had severe or critically severe symptoms, accounting for 54.8%. The mean age of severe patients is 74 (ranging from 52 to 91) and 69.14 (ranging from 58 to 82) of non‐severe patients. In this study, a majority of patients (74.4%, 29/39) were male and most patients (87.2%, 34/39) were over 60 years, consistently with previous literature reports.^15^, ^16^

**FIGURE 1:**
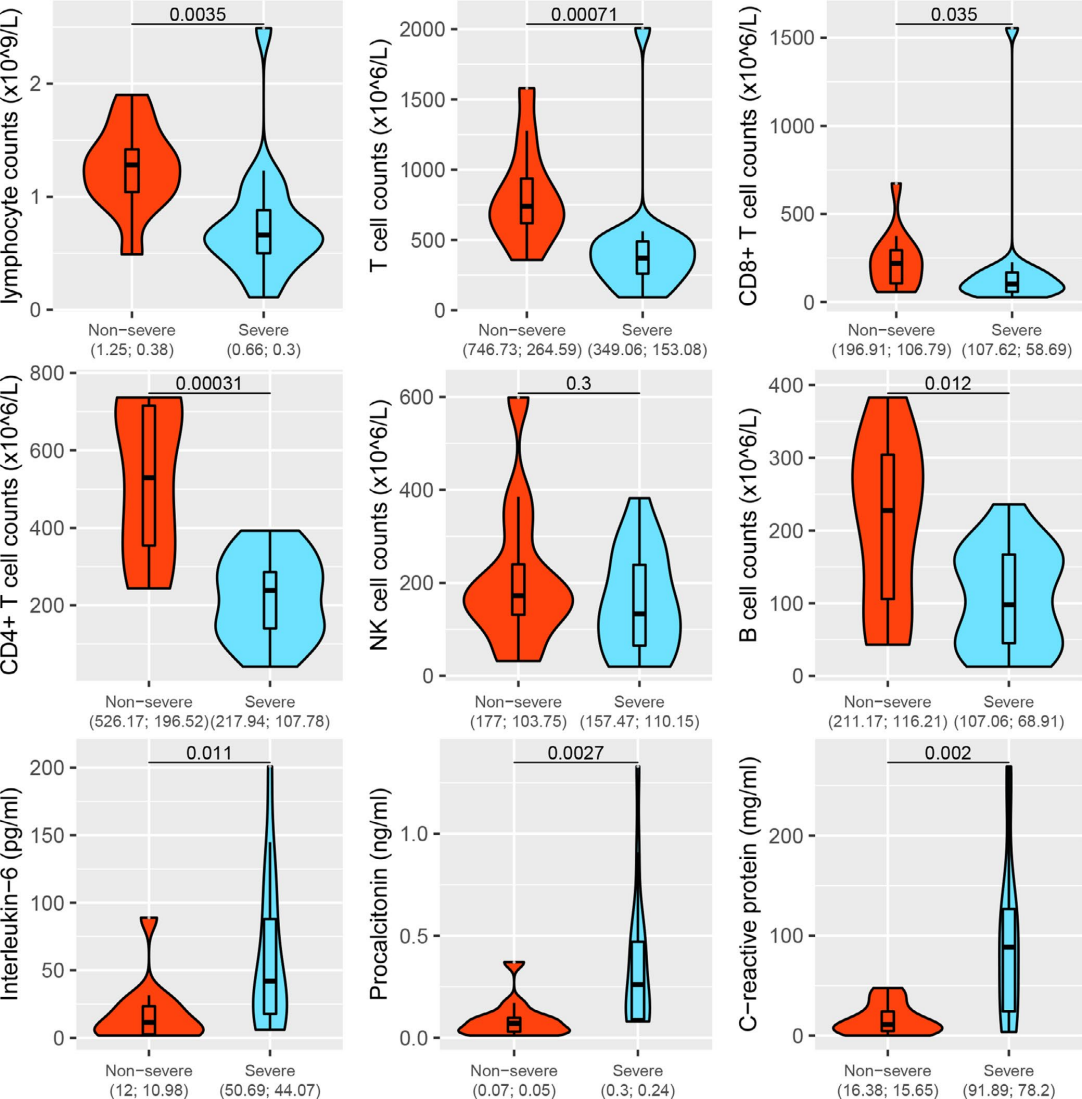
Comparison of immune status between severe and non‐severe patients

For the immune status, we observed several immune cells showed drastic alteration whereas disease progressed from non‐severe condition to severe condition. A significant decrease in total lymphocytes (*P* =0.0035), absolute number of total T cells (*P* =0.00071), Total CD8 + T lymphocyte count (*P* =0.035), absolute number of CD4 + T cells (*P* =0.00031), absolute number of B cells (*P* =0.012), and a significant increase in IL‐6 (*P* =0.011), Procalcitonin (*P* =0.0027) and CRP (*P* =0.002) was observed (Figure 1). Other indexes like proportion of total T cells and proportion of cytotoxic T lymphocyte revealed no significant difference between two groups in our study. These results suggested different immune response of different clinical severity of patients, similar to previous reports.^17^, ^18^

### 3.2 Altered lncRNAs between severe vs. non‐severe patients

**FIGURE 2:**
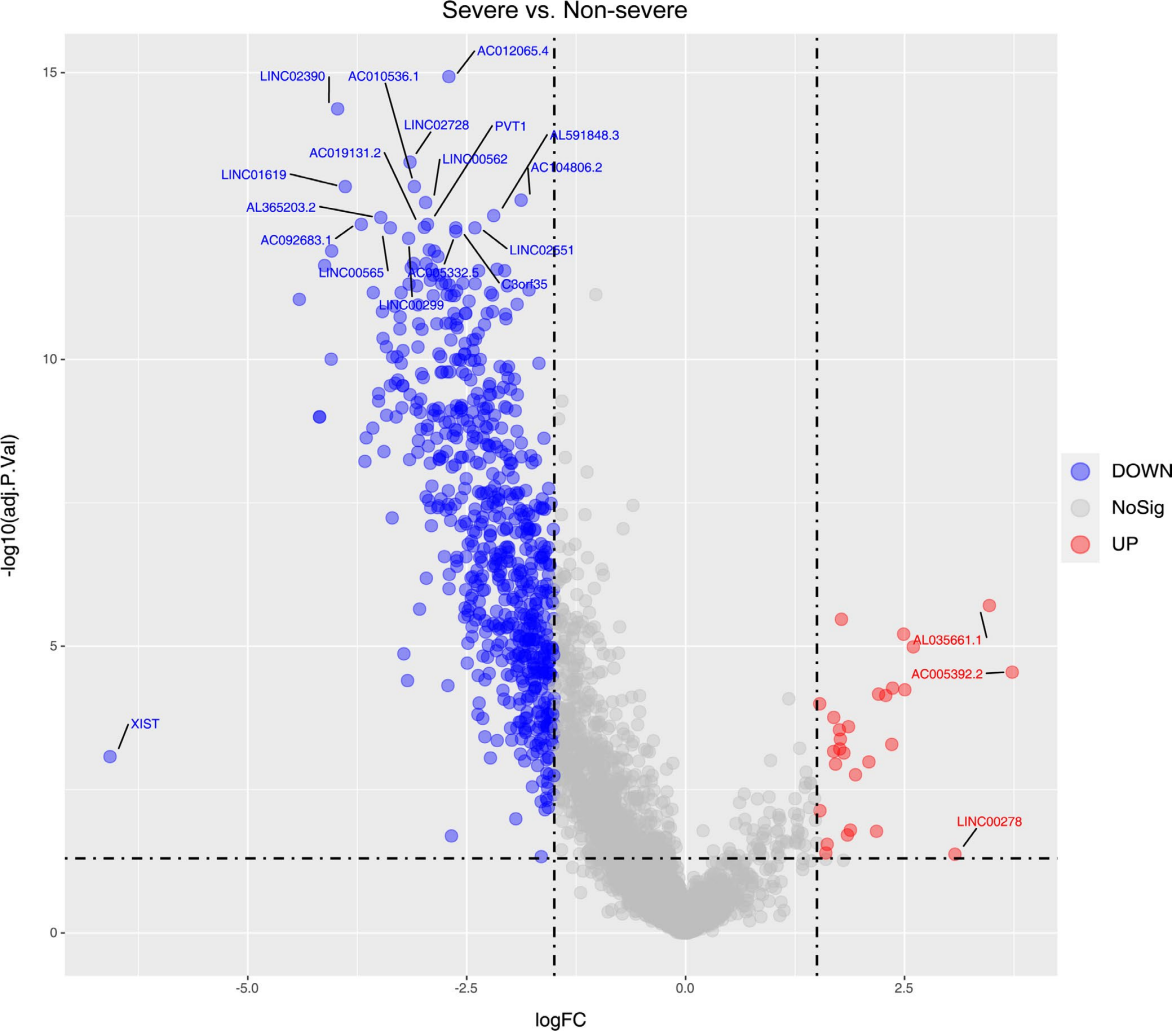
Volcano plot of differentially expressed lncRNAs between severe and non‐severe COVID‐19 patients

To profile the peripheral lncRNA signature response to COVID‐19, we performed a transcriptional analysis of lncRNAs using RNA‐Seq. A spectrum of 2511 functionally active lncRNAs was identified by utilizing stringent criteria (RPKM in at least 10% of samples). The comparison between the severe and non‐severe COVID‐19 patients was demonstrated in a volcano plot (Figure 2). Overall, we found 687 lncRNAs using the criteria of *P*‐value < 0.05 and |log2(fold change)|>=1 (Table S1). A number of most significantly regulated lncRNA signatures were listed in Table 1. Among the up‐regulated lncRNAs, 3 lncRNAs had a log fold change above 3. Among the down‐regulated lncRNAs, 1 lncRNA had a log fold change above 6 and 6 lncRNAs had a fold change between 4 and 6. The most up‐regulated one was ENSG00000231412 (AC005392.2) (log fold change: 3.73), followed by ENSG00000274173 (AL035661.1) (log fold chang: 3.47) and ENSG00000231535 (LINC00278) (log fold change: 3.08). The most down‐regulated one was ENSG00000229807 (XIST) (log fold change: −6.573), followed by ENSG00000273160 (AL359962.2) (log fold change: −4.412). Also, the expression quantity of lncRNA between patients and healthy controls was compared (Figure S1 and Figure S2). Venn diagram was also used to summarize the finding (Figure S3). Overall, there were 1072 lncRNAs differentially expressed in both groups. With the exclusion of the lncRNAs only differentially expressed in both two groups, there were 215 specific lncRNAs differentially expressed in three groups.

**TABLE 1:** Top 10 up‐ and down‐regulated LncRNA in severe patients when compared with non‐severe patients

Down‐regulated LncRNAs							
ensembl‐ID	logFC	AveExpr	*t*	*P* value	adj.P. Val	B	Symbol
ENSG00000229807	−6.573	1.542	−4.149	1.86E‐04	8.42E‐04	0.092	XIST
ENSG00000273160	−4.412	2.669	−11.368	1.13E‐13	8.98E‐12	21.097	AL359962.2
ENSG00000223511	−4.180	0.081	−9.204	3.92E‐11	1.01E‐09	15.290	AL683807.1
ENSG00000223511	−4.180	0.081	−9.204	3.92E‐11	1.01E‐09	15.290	AL683807.1
ENSG00000256427	−4.123	−0.124	−12.225	1.32E‐14	2.31E‐12	23.224	AC010175.1
ENSG00000270069	−4.048	0.240	−10.236	2.24E‐12	9.91E‐11	18.136	MIR222HG
ENSG00000276867	−4.042	−0.337	−12.561	5.82E‐15	1.29E‐12	24.032	AC074050.4
ENSG00000256582	−3.974	0.819	−16.160	2.04E‐18	4.28E‐15	31.836	LINC02390
ENSG00000257242	−3.887	1.620	−14.255	1.15E‐16	9.70E‐14	27.892	LINC01619
ENSG00000230606	−3.704	2.173	−13.267	1.09E‐15	4.42E‐13	25.682	AC092683.1

Up‐regulated LncRNAs							
ensembl‐ID	logFC	AveExpr	*t*	*P* value	adj.P. Val	B	Symbol
ENSG00000231412	3.729	1.059	5.372	4.36E‐06	2.84E‐05	3.758	AC005392.2
ENSG00000274173	3.469	−0.177	6.338	2.13E‐07	1.95E‐06	6.742	AL035661.1
ENSG00000231535	3.075	0.197	2.531	1.57E‐02	4.25E‐02	‐4.104	LINC00278
ENSG00000273812	2.600	0.558	5.737	1.39E‐06	1.03E‐05	4.883	BX640514.2
ENSG00000283036	2.503	−1.086	5.119	9.59E‐06	5.76E‐05	2.983	LINC01988
ENSG00000275527	2.489	0.588	5.922	7.81E‐07	6.16E‐06	5.456	AC100835.2
ENSG00000261172	2.363	0.503	5.143	8.92E‐06	5.39E‐05	3.055	AC133919.2
ENSG00000237520	2.354	−1.232	4.332	1.07E‐04	5.16E‐04	0.623	AL391832.1
ENSG00000226012	2.285	0.014	5.042	1.22E‐05	7.24E‐05	2.747	AP001434.1
ENSG00000249790	2.203	6.121	5.060	1.15E‐05	6.85E‐05	2.804	AC092490.1

### 3.3 Hierarchical clustering and PCA

**FIGURE 3:**
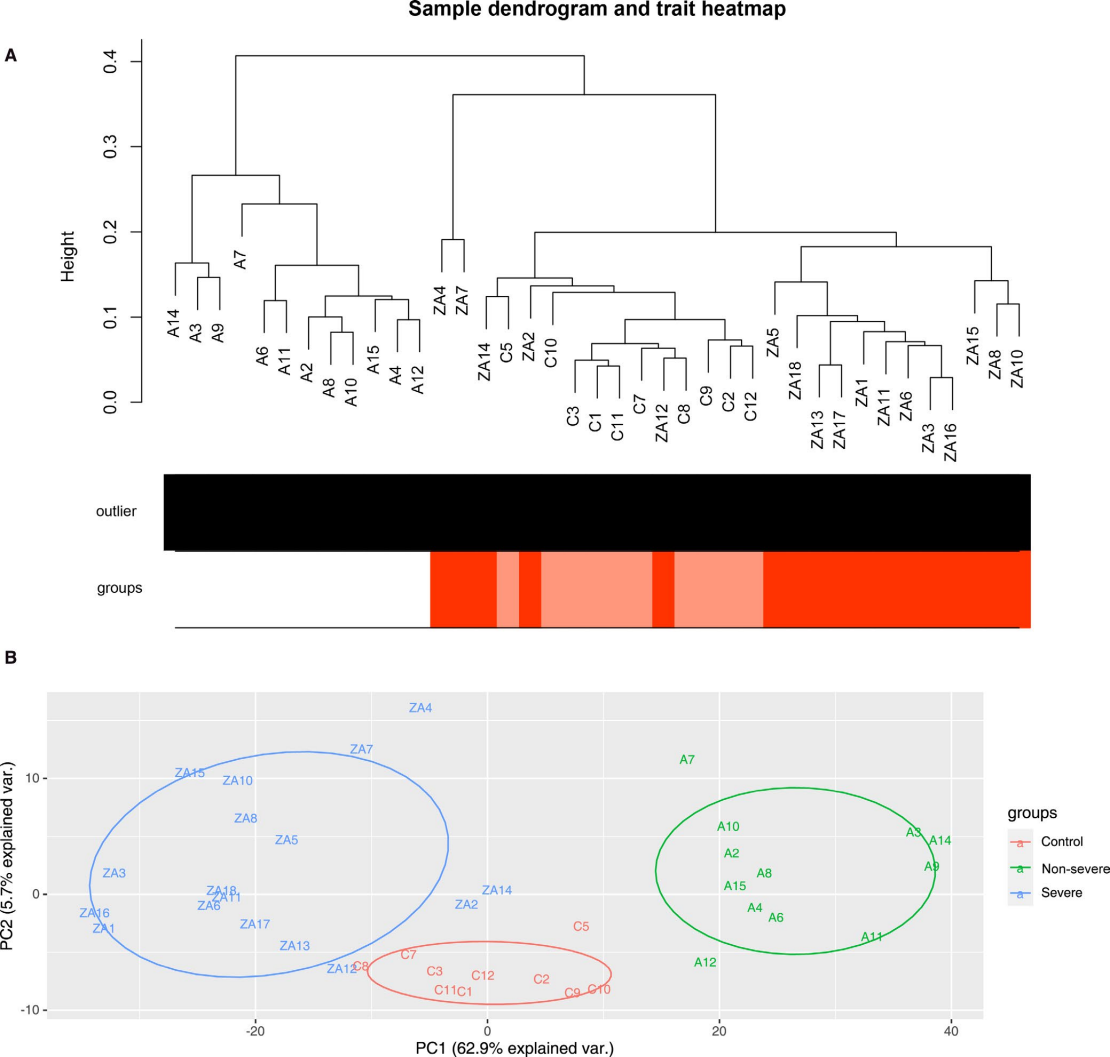
(A) Hierarchical clustering conducted on CuffDiff2 expression values (in FPKM). ZA/A/C are clearly separated in two distinct groups. (B) PCA of variant genes in which input samples are clustered in severe patients (blue circles), non‐severe patients (green circles) and control (red circles) groups

We calculated RNA‐seq based lncRNA expression levels in FPKM and performed unsupervised hierarchical clustering to detect similarities in gene expression profiles between severe, non‐severe and healthy control by fashClust function of the WGCNA package (Figure 3A). We observed an obvious separation between those three groups, suggesting specific lncRNA profile signatures for COVID‐19 patients. Principal component analysis (PCA) of variant genes re‐marked such separation (Figure 3B). PCA was able to distinguish severe patients demonstrated that the severe patients separated from the healthy control and non‐severe patients (PC1: 62.9%, PC2: 5.7%). This result enhanced that the lncRNA profile of severe patients differs a lot from non‐severe patients and healthy control.

### 3.4 Development of weighted co‐expression network and identification of key modules

**FIGURE 4:**
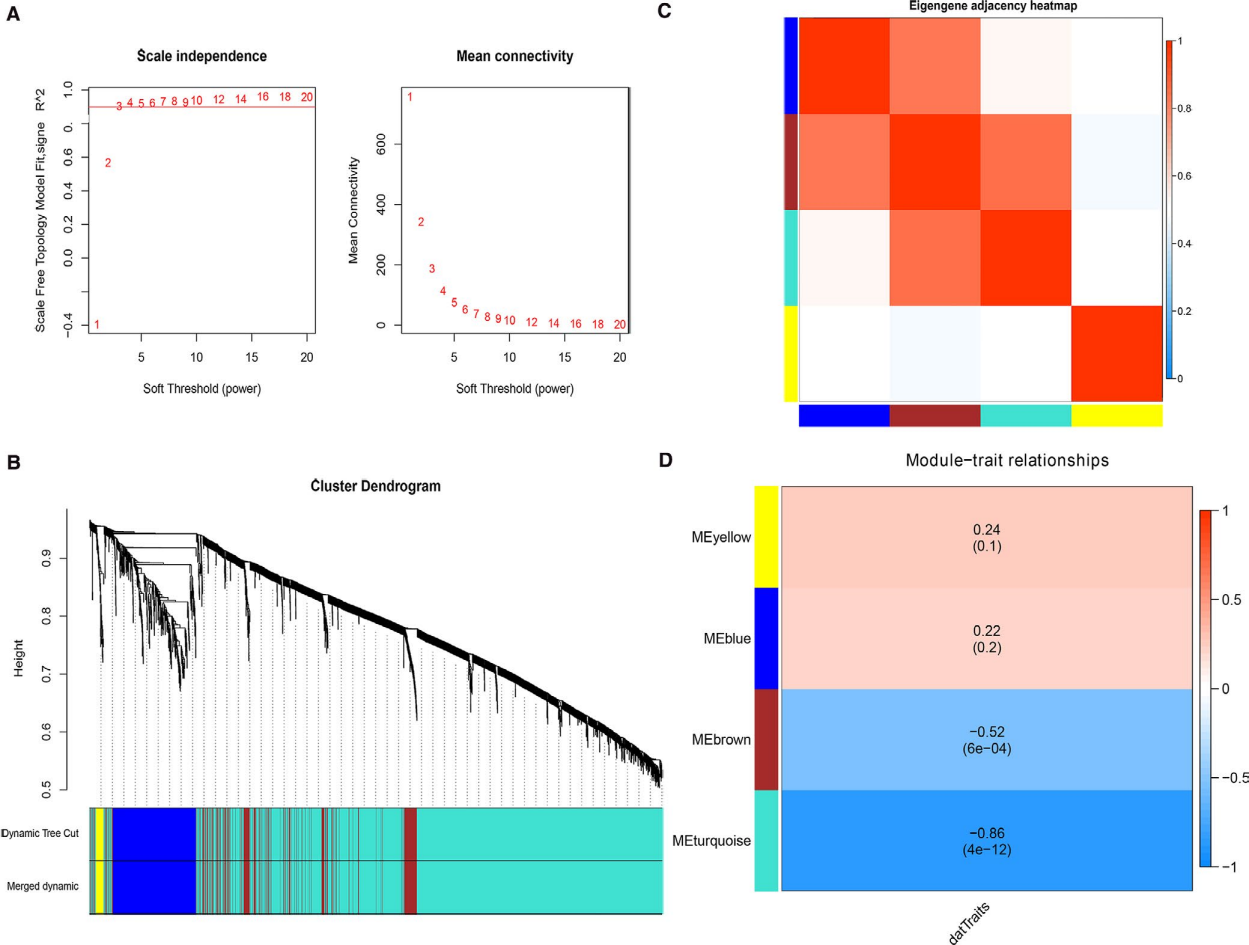
(A) Soft threshold in Weighted Gene Co‐Expression Network Analysis (WGCNA) of lncRNAs. (B) Using topological overlapping matrix dissimilarity, the cluster dendrogram was prepared to show four individual modules including MEyellow, MEblue, MEbrown and MEturquoise. (C) Heat map plot of the eigenegene adjacencies in the network; (D) the correlation analysis between the four modules and severity level. The ME brown and MEturquoise module shows negative correlation with the severity phenotype

Selection of the soft‐thresholding power is an important step when constructing a WGCNA. We performed the analysis of network topology for thresholding powers from 1 to 20 and identified the relatively balanced scale independence and mean connectivity of the WGCNA. As shown in Figure 4A and B, power value 3, which was the lowest power for the scale‐free topology fit index on 0.9, was selected to produce a hierarchical clustering tree (dendrogram) of lncRNAs. We set the MEDissThres as 0.25 to merge similar modules. Finally, 4 modules were identified. There were 366 lncRNA in the blue module, 34 lncRNA in the yellow module, 258 lncRNA in the brown module and 1862 lncRNAs in the turquois module.

Interaction relationships of the 4 modules were analysed, and the network heat map was plotted (Figure 4C). The results revealed that each module was an independent validation to each other, which demonstrated a high level of independence among the modules and relative independence of genes expression in each module. Importantly, we calculated the correlation of the modules and the severity of disease. The correlation of the blue module, turquoise module, brown module and yellow module was 0.22, −0.86, −0.52 and 0.24, respectively. The p‐values of brown and turquoise module are significant (*P*‐value < 0.01), which indicated that the brown and turquoise module may play an important role in the development of the COVID‐19 (Figure 4D). We identified the turquoise module and brown module as the modules most relevant to the disease severity.

### 3.5 lncRNA subtypes categorize COVID‐19 patients based on iCluster algorithm

**FIGURE 5:**
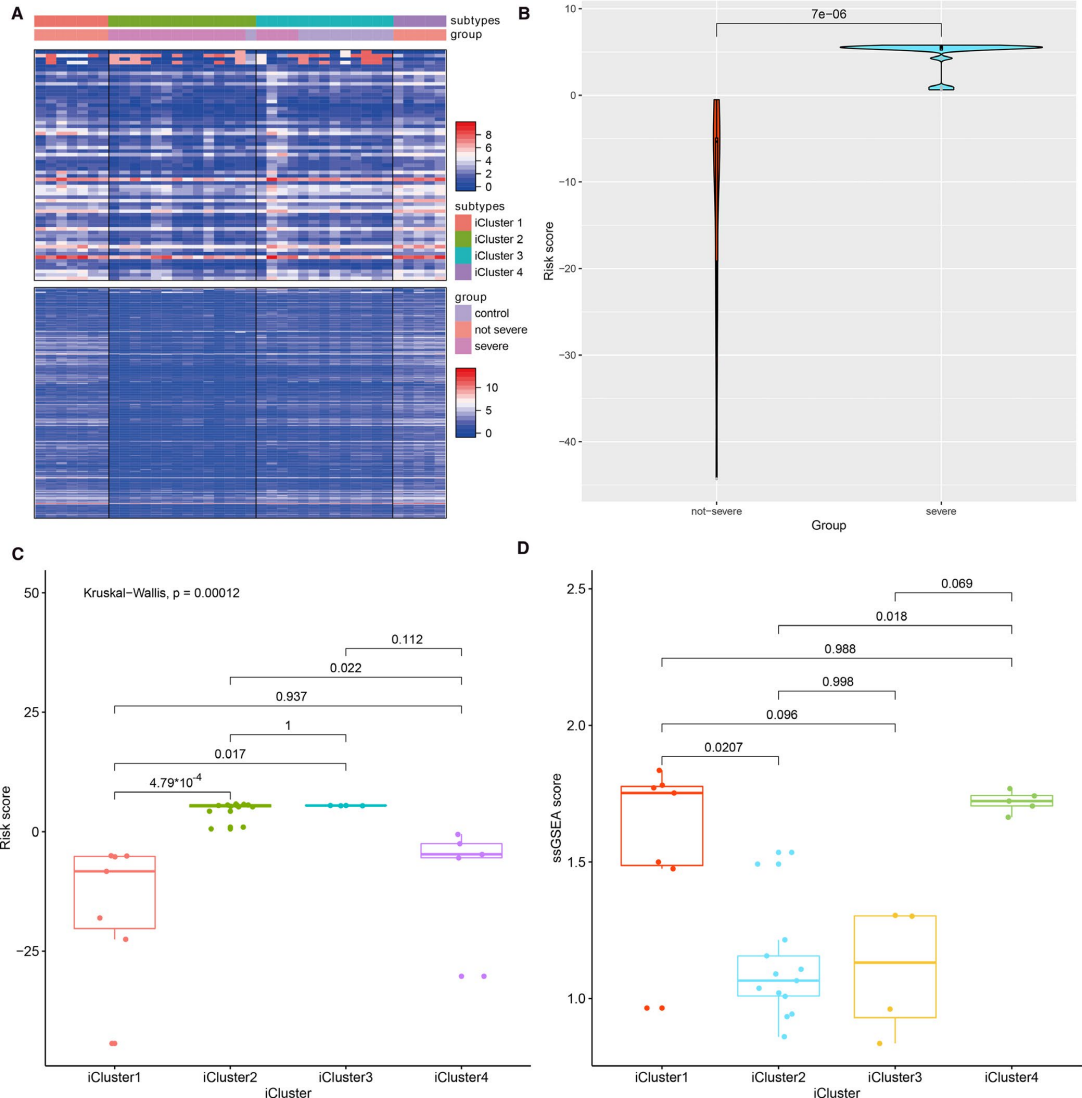
(A) Four clusters of lncRNAs were identified based on icluster algorithm. (B) Risk score by lncRNA pattern was compared between severe and non‐severe patients. (C) Risk score was compared in four icluster subtypes. (D) Immune score was compared in four lncRNA subtypes

The optimal combination of subtypes was determined via minimizing a Bayesian Information Criterion (BIC). An ‘elbow’ point was noted at K = 3, beyond which the BIC kept increasing and thus the 4‐class solution was chosen (Figure S4). In total, four subtypes were identified (Figure 5A). The outcome of iClusters algorithm showed that COVID‐19 is a heterogeneous disease with multi‐subtypes. The signature of each iCluster was shown in Table [Link], [Link], [Link], [Link]. We inferred that patients in different subtypes can represent different severe conditions. Thus, we developed a LASSO model to calculate patients’ risk scores. The risk score for predicting severity of COVID‐19 patients was calculated based on the expression levels and the corresponding regression coefficients of the 7 lncRNAs (Table 2). Based on the risk score, we observed a significant difference between severe and non‐severe COVID‐19 patients (*P* = 7e‐6) (Figure 5B). Subsequently, the area under the curve (AUC) value of the receiver operating characteristic (ROC) curve revealed the significant performance of this 7‐lncRNAs signature in differentiating the severe and non‐severe patients (AUC = 1) (Figure S5).

**TABLE 2:** The detailed information of the 7‐lncRNA pattern predicting risk of severity in COVID‐19 patients

Ensemble ID	Gene symbol	Gene type	Chromosome location
ENSG00000272002	AC010904.2	lincRNA	chr2:7260871‐7261504:(+)
ENSG00000270100	AC012065.4	lincRNA	chr2:20678254‐20678932:(‐)
ENSG00000273038	AL365203.2	lincRNA	chr10:32887255‐32889311:(‐)
ENSG00000256427	AC010175.1	lincRNA	chr12:9246497‐9257960:(+)
ENSG00000260388	LINC00562	lincRNA	chr13:47930153‐47932622:(‐)
ENSG00000226180	AC010536.1	lincRNA	chr16:87693537‐87696147:(‐)
ENSG00000266288	AP005671.1	lincRNA	chr18:5381641‐5385330(‐)

We compare the possible risk score in every subtype and observed that severe patients have two different subtypes (iCluster 2 and iCluster 3) with indicate a clear subtype in severe patients (Figure 5C). Next, we compared immune status between different iClusters subtypes. We calculated the immune scores to measure the immune status of patients. The signatures of each immune index which was significant different between non‐severe and severe patients were screened using Bortua R package. The immune scores were calculated based on all signatures utilizing GSVA R package. We conclude that subtype of iCluster 2 and iCluster 3 had a significant low immune score when compared to iCluster 1 and iCluster 4, consistent with the severe clinical condition (Figure 5D).

## 4 DISCUSSION

Long non‐coding RNA (lncRNA) is a subclass of endogenous, non‐protein‐coding RNA, which lacks an open reading frame and is more than 200 nucleotides in length. As the lncRNAs expression pattern remains unclear in COVID‐19 patients, our study is the first analysis to provide detailed lncRNAs information as molecular biomarkers. Here, we used RNA sequencing to characterize the lncRNA expression pattern in peripheral blood from 17 severe patients and 12 non‐severe patients and 10 healthy controls. Overall, we observed that many lncRNAs were significantly altered as we compared between the three groups: severe, non‐severe COVID‐19 patients and healthy control. For example, we observed lncRNA GATA5 was significantly elevated in severe condition. In a paper published by Gennadi, GATA5 was repressor of ACE2 gene which may be putative mitigation agents.^19^

The driving factors in COVID‐19 remain unclear. It is urgent for the doctors to identify from the numerous outpatients as soon as possible who will develop severe conditions like ARDS or even death. In a paper published by Zhou et al, the authors confirmed the older age, high SOFA score and d‐dimer greater than 1μg/mL are potential risk factors by logistic regression methods from 191 patients with 54 deaths.^20^ T cell and B cell functions decreased with age. The excess production of type 2 cytokines could lead to a deficiency in control of viral replication and produce more prolonged pro‐inflammatory responses, potentially leading to poor outcome.^21^ However, as the cases are climbing rapidly among young adults, for example, in California, more than 44% of new diagnoses are in people age 34 or younger, it is urgent for us to dig out more markers which can predict the severity of the COVID‐19 patients. One major finding of our study is that we reported a good 7‐lncRNA panel which can represent the severity of the disease with the area under curve of 1 for the first time. These 7 lncRNAs, AC010904.2, AC012065.4, AL365203.2, AC010175.1, LINC00562, AC010536.1 and AP005671.1, were firstly reported in COVID‐19 patients. However, the detailed biological mechanism of these lncRNAs is missing which needs more investigation. Although the biological importance of these unreported lncRNAs need further evaluation by experiments, our study proposed an efficient strategy to identify important lncRNAs associated with COVID‐19 and predict their potential functional roles, which may guide subsequently mechanism experiments.

From immune index of these patients, we observed a significant decrease index like total lymphocytes, T cell counts, CD4 + T cell counts and B cell counts and a significant increase in Procalcitonin and CRP. The results were consistent with previous studies. Chen et al characterized the immunological features of COVID‐19 patients with different severity of the disease. Totally, 11 patients with severe disease displayed significantly higher serum levels of IL‐6, IL‐10 and TNF‐α and lower absolute numbers of T lymphocytes, CD4 + T cells and CD8 + T cells as compared with 10 moderate patients.^22^ Moreover, Xiong et al performed a transcriptome sequencing analysis of several pro‐inflammatory genes in both PMCs and bronchoalveolar lavage fluid of patients with COVID‐19, compared with samples from healthy donors. Laboratory findings of the 3 patients indicated cell count reduction of various types of immune cells including lymphocytes in patients’ blood.^23^ Another study by Wang et al, including 65 SARS‐CoV‐2‐positive patients, showed that the absolute numbers of CD4 + T cells, CD8 + T cells and B cells progressively decreased in relation with increasing severity of illness.^24^ All these evidence have been demonstrated that disease progression of COVID‐19 is dominated by the progressive loss of lymphocytes and gain of myeloid cells. The ‘cytokine storm’ and the subsequent ARDS result from the effects of the combination of several immune‐active molecules. The ‘cytokine storm’ is the most dangerous and potentially life‐threatening event related to COVID‐19.^25^ In recent years, the physiological roles of lncRNAs in immune system emerge as vital and inspiring. For example, lncRNA‐EPS can precisely regulate in macrophages to control the expression of immune response genes.^26^ Zhang et al also reported that lncRNA NEAT1 can significantly reduce the expression of a group of chemokines and cytokines, including IL‐6, CXCL10.^27^ In our study, we extracted total RNA from peripheral blood of each patients, so the lncRNA expression pattern from these RNAs can be regarded as the unique feature representing the severity condition of these patients, regardless of the specific PBMC types. Based on these immune indexes, we develop an immune score which can represent the patient's immune status by GSVA algorithm based on these lncRNA signatures. It is of vital significance to further investigate the lncRNAs’ relationship with the immune status.

Some studies explored the lncRNAs in COVID‐19 patients. Vishnubalaji et al revealed 155 up‐regulated and 195 down‐regulated lncRNAs comparing SARS‐CoV‐2 infected bronchial epithelial cells with normal human bronchial epithelial cells.^9^ Yousefi et al also to determine the importance of key non‐coding RNAs involving in TGF‐beta signalling pathway as potential therapeutic targets by computational analysis.^10^ However, none of them reported featured lncRNAs between severe and non‐severe COVID‐19 patients especially for risk stratification. By iCluster algorithm methods, our study is the first analysis to provide lncRNA‐based stratification of COVID‐19 patients by means of lncRNA subtypes. The proposed subtypes are characterized by distinct molecular profiles defined by lncRNA expression. The icluster‐based subtypes are associated with the severity of the disease and immune status. In particular, it is evident that subtype iCluster1 and iCluster4 is associated with more favourable condition of COVID‐19 and subtype iCluster2 and iCluster3 indicates more severe condition. It is our surprising finding that in severe patients, we observe two subtypes (iCluster2 and iCluster3) with similar immune scores and risk scores. It indicates that severe patients can be divided into two subtypes by lncRNA expression pattern. It is of great value for us to further analyse the differed lncRNAs in the prediction of severity of COVID‐19.

One disadvantage of this study is that the data limit our ability to further analyse the function of these lncRNAs. We plan to compare lncRNA expression‐based subtypes with mRNA expression‐based subtypes in further study. Moreover, we hope more studies can focus on the risk prediction or prognosis ability of lncRNAs with a larger number of COVID‐19 patients in the future.

In summary, we have presented a lncRNA atlas of the peripheral immune response to COVID‐19. These data highlight immunological features associated with severity of the disease. These lncRNAs can be new surrogate biomarker of diagnosis and prognosis in vitro of COVID‐19 patients.

## 5 CONCLUSIONS

In conclusion, we have identified a substantial number of COVID‐19 related lncRNAs in this study, and we have imputed potential immunological functions for them in the pathogenesis of COVID‐19 patients. Moreover, our results provide interesting potential clues into the mechanisms of lncRNA panel in the severity of COVID‐19 ARDS. As the roles of lncRNAs in COVID‐19 have not yet been fully identified and understood, this analysis should provide valuable resource and information for the future studies.

## 6 CONFLICT OF INTEREST STATEMENT

The authors confirm that there are no conflicts of interest.

## AUTHOR CONTRIBUTION

Jie Cheng: Conceptualization (equal); Data curation (equal); Formal analysis (equal); Funding acquisition (equal); Investigation (equal); Methodology (equal); Software (equal); Supervision (equal); Validation (equal); Writing‐original draft (equal); Writing‐review & editing (equal). Xiang Zhou: Conceptualization (equal); Investigation (equal); Validation (equal); Writing‐original draft (equal). Weijun Feng: Formal analysis (equal); Validation (equal); Writing‐original draft (equal); Writing‐review & editing (equal). Min Jia: Investigation (supporting); Methodology (supporting); Writing‐original draft (supporting). Xinlu Zhang: Data curation (supporting); Formal analysis (supporting); Writing‐original draft (supporting). Taixue An: Formal analysis (supporting); Software (supporting); Writing‐original draft (supporting). Minyuan Luan: Software (equal); Writing‐review & editing (equal). Yi Pan: Software (equal); Writing‐original draft (supporting). Shu Zhang: Methodology (supporting); Validation (supporting). Zhaoming Zhou: Conceptualization (supporting); Data curation (supporting); Software (supporting). Lei Wen: Writing‐original draft (supporting); Writing‐review & editing (supporting). Yun Sun: Conceptualization (lead); Funding acquisition (lead); Writing‐review & editing (lead). Zhou Cheng: Funding acquisition (lead); Project administration (lead); Writing‐original draft (lead); Writing‐review & editing (lead).

## Supporting information

Fig S1

Fig S2

Fig S3

Fig S4

Fig S5

Table S1

Table S2

Table S3

Table S4

Table S5
