# Supplementary figures and images for: Risk stratification by long non‐coding RNAs profiling in COVID‐19 patients
Source: J Cell Mol Med. 2021 Mar 23;25(10):4753–64. doi: 10.1111/jcmm.16444 (PMC8107096; doi:10.1111/jcmm.16444)

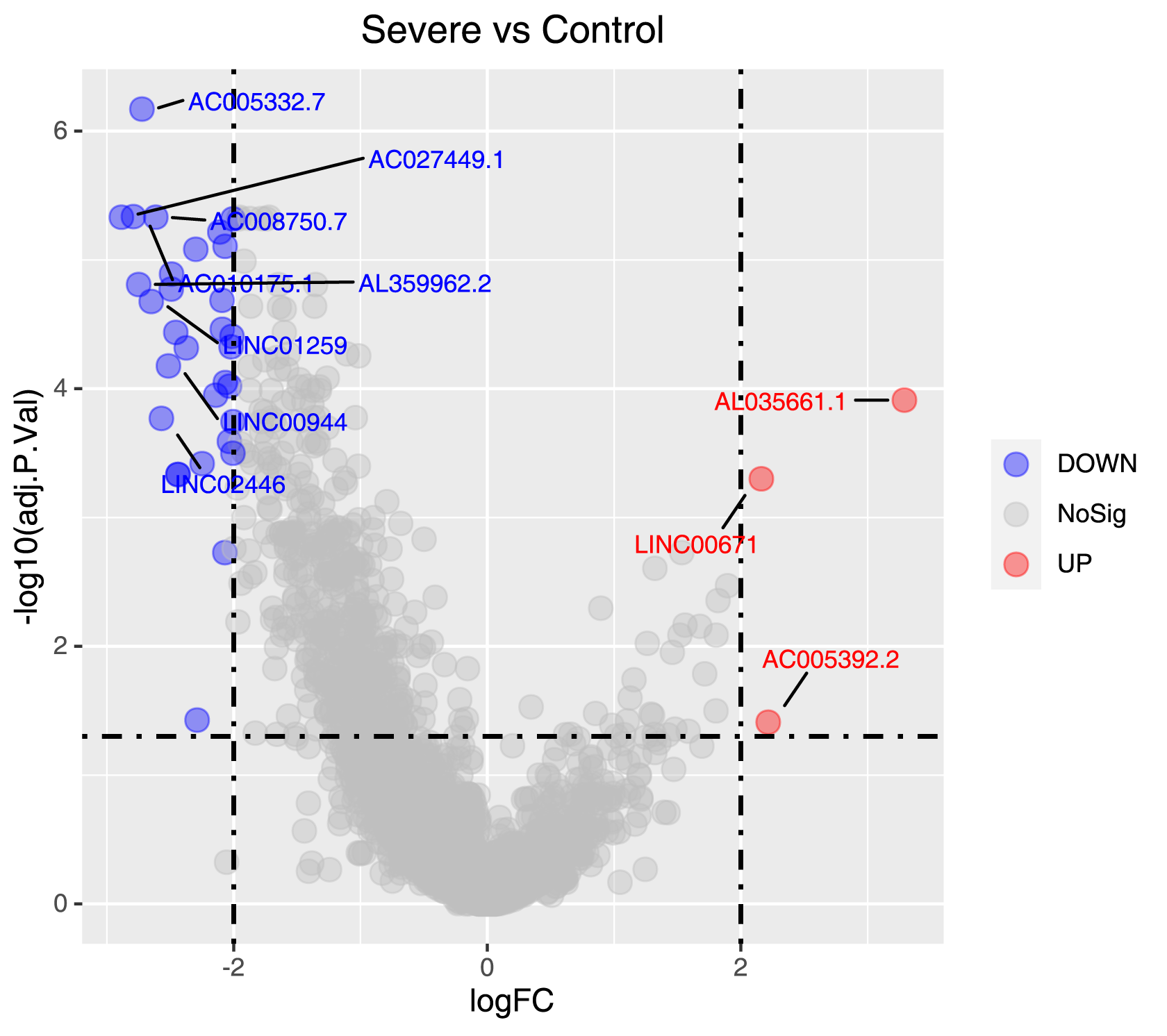

Supplement: Supplementary file 1 — Fig S1 [file JCMM-25-4753-s008.tif]

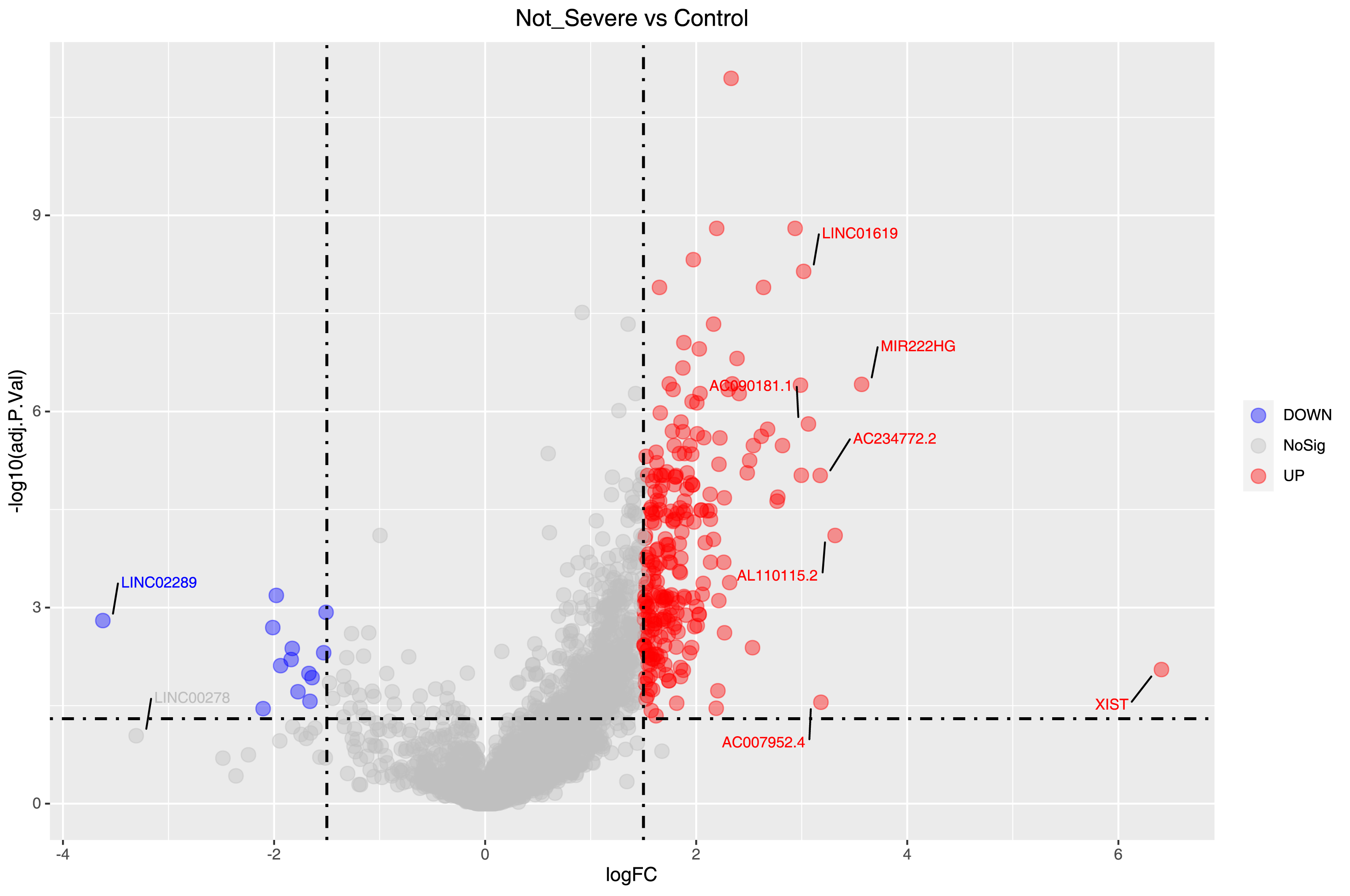

Supplement: Supplementary file 2 — Fig S2 [file JCMM-25-4753-s010.tif]

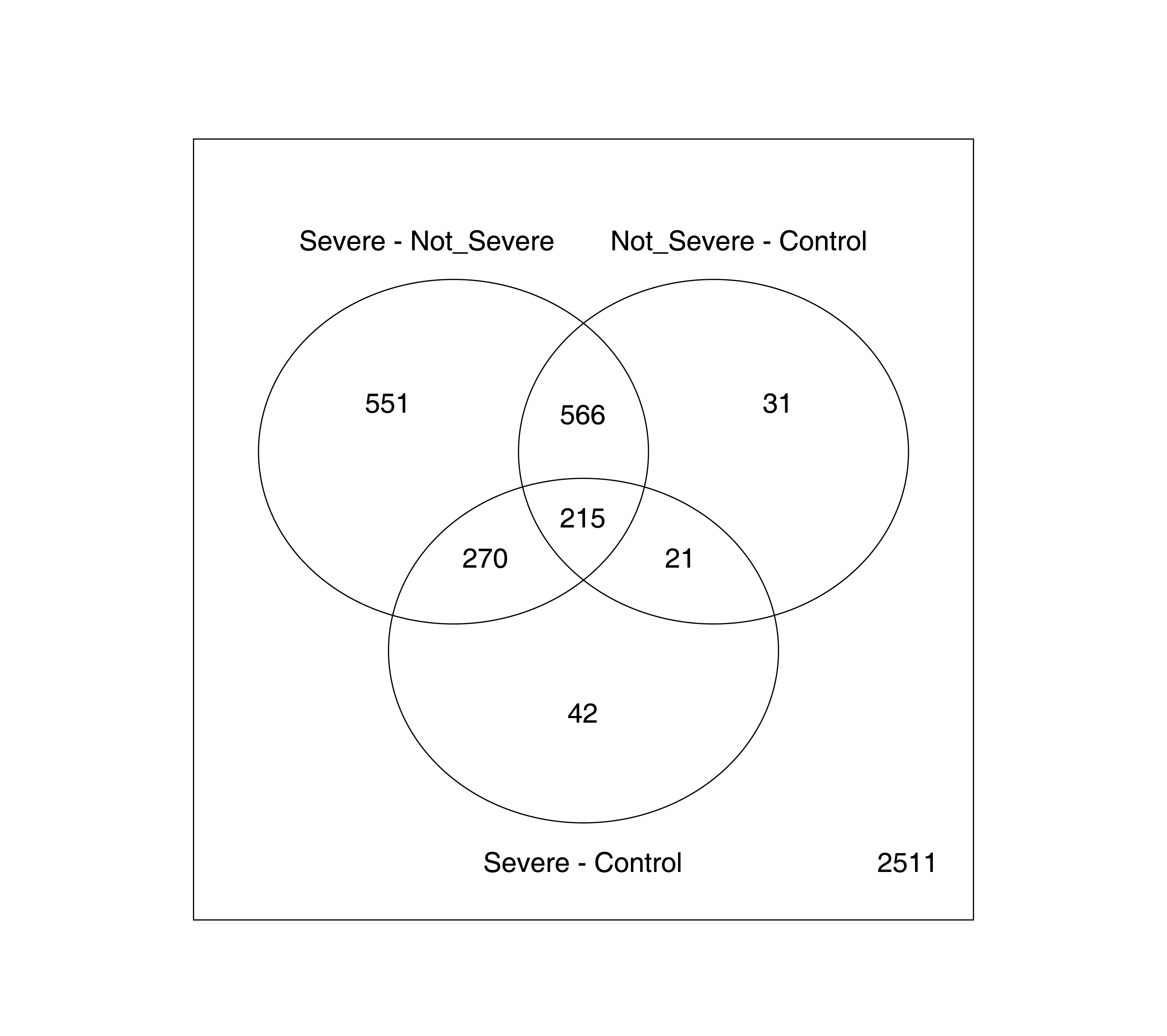

Supplement: Supplementary file 3 — Fig S3 [file JCMM-25-4753-s006.tif]

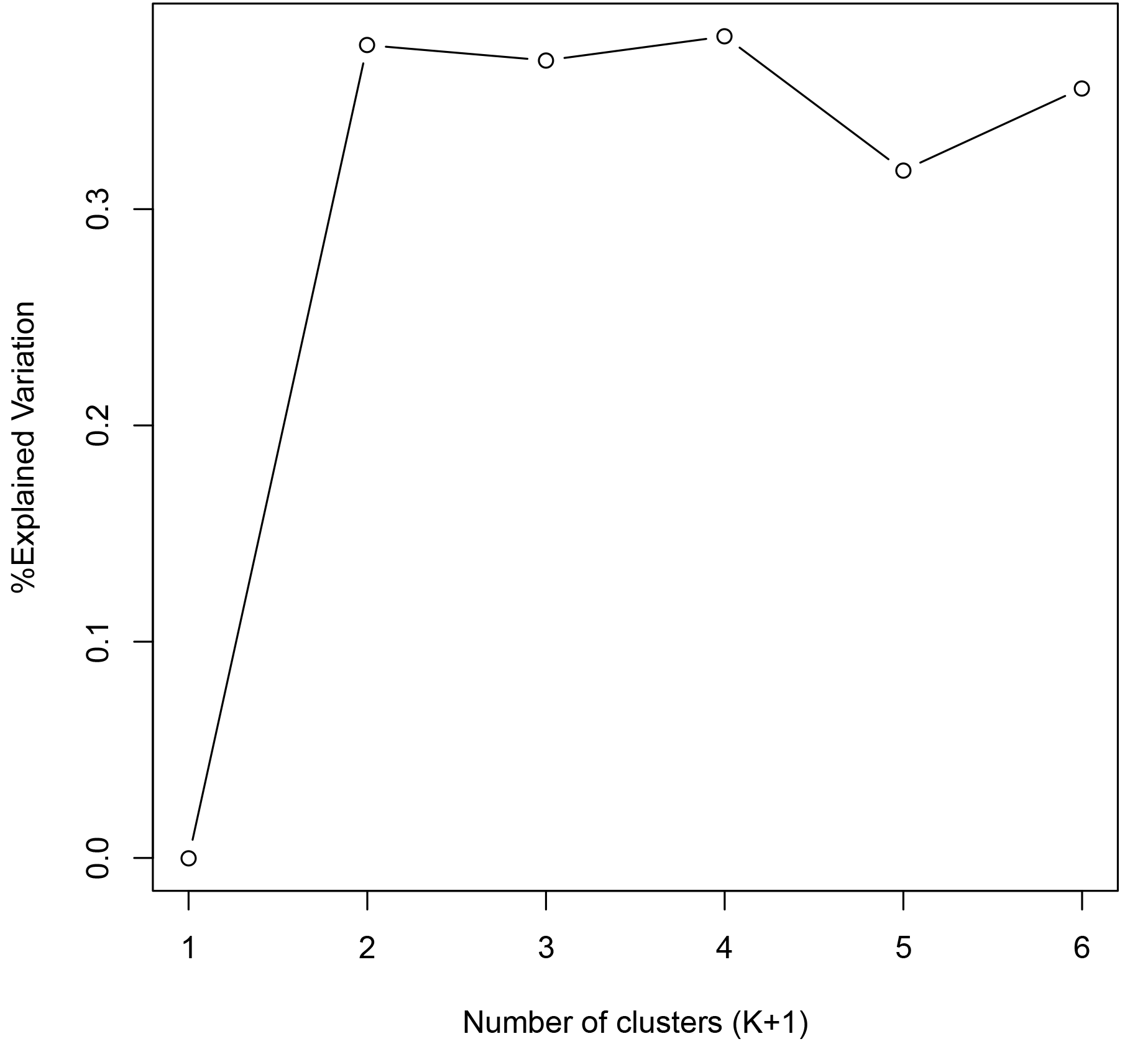

Supplement: Supplementary file 4 — Fig S4 [file JCMM-25-4753-s004.tif]

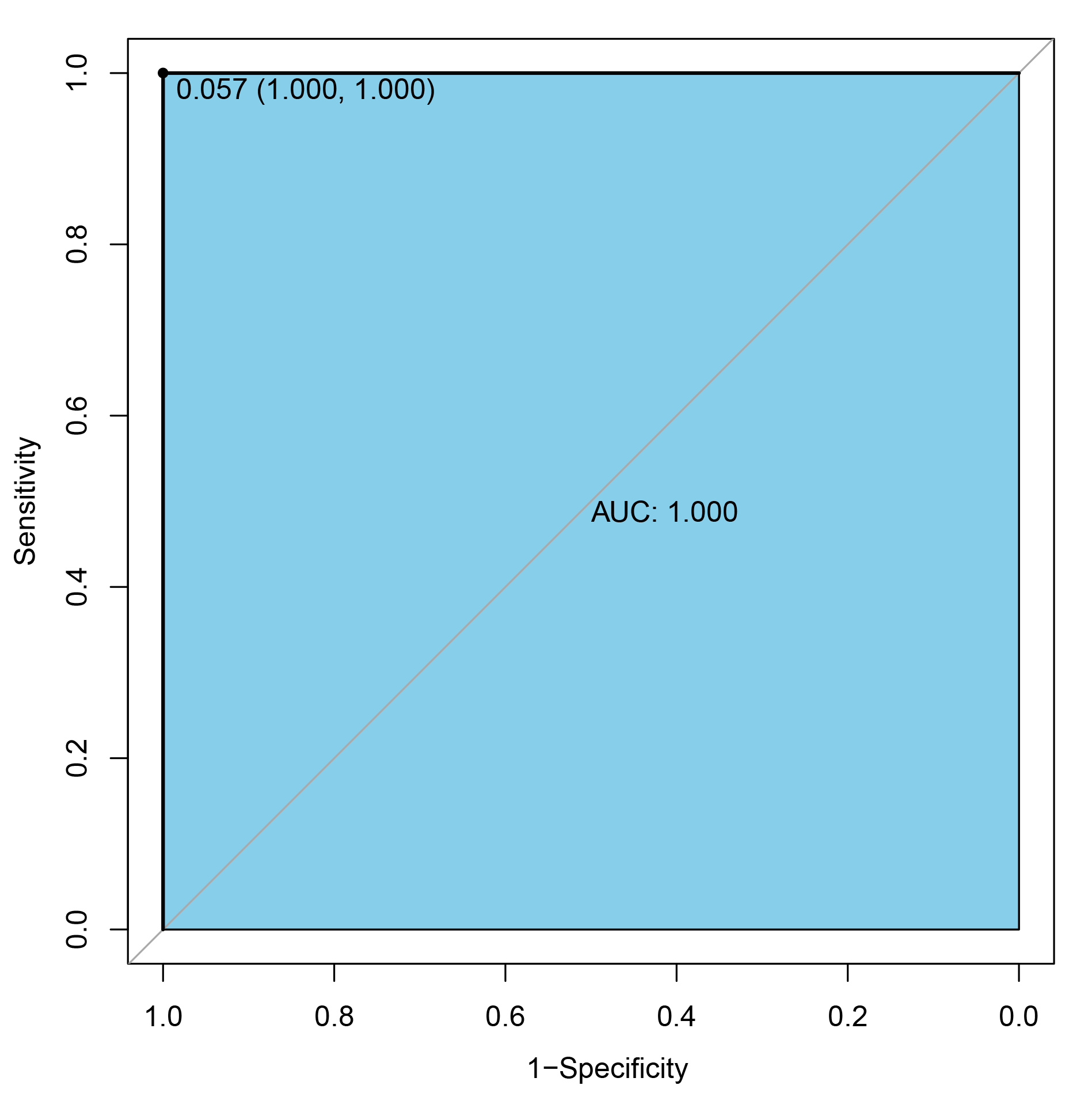

Supplement: Supplementary file 5 — Fig S5 [file JCMM-25-4753-s005.tif]
